# The Role of Individual Traits and Environmental Factors for Diet Composition of Sheep

**DOI:** 10.1371/journal.pone.0146217

**Published:** 2016-01-05

**Authors:** Atle Mysterud, Gunnar Austrheim

**Affiliations:** 1 Centre for Ecological and Evolutionary Synthesis (CEES), Department of Biosciences, University of Oslo, P.O. Box 1066 Blindern, NO-0316, Oslo, Norway; 2 Museum of Natural History and Archaeology, Section of Natural History, Norwegian University of Science and Technology, NO-7491, Trondheim, Norway; The University of Wollongong, AUSTRALIA

## Abstract

Large herbivore consumption of forage is known to affect vegetation composition and thereby ecosystem functions. It is thus important to understand how diet composition arises as a mixture of individual variation in preferences and environmental drivers of availability, but few studies have quantified both. Based on 10 years of data on diet composition by aid of microhistological analysis for sheep kept at high and low population density, we analysed how both individual traits (sex, age, body mass, litter size) linked to preference and environmental variation (density, climate proxies) linked to forage availability affected proportional intake of herbs (high quality/low availability) and *Avenella flexuosa* (lower quality/high availability). Environmental factors affecting current forage availability such as population density and seasonal and annual variation in diet had the most marked impact on diet composition. Previous environment of sheep (switch between high and low population density) had no impact on diet, suggesting a comparably minor role of learning for density dependent diet selection. For individual traits, only the difference between lambs and ewes affected proportion of *A*. *flexuosa*, while body mass better predicted proportion of herbs in diet. Neither sex, body mass, litter size, ewe age nor mass of ewe affected diet composition of lambs, and there was no effect of age, body mass or litter size on diet composition of ewes. Our study highlights that diet composition arises from a combination of preferences being predicted by lamb and ewes’ age and/or body mass differences, and the immediate environment in terms of population density and proxies for vegetation development.

## Introduction

Large herbivore foraging is known to affect vegetation composition and hence ecosystem function [1,2]. Understanding what causes variation in the diet of large herbivores is therefore important [3], and also provides a link to their own performance [4]. Nutritional quality and sward structure are main determinants of preference [5]. Nutritional quality ranks first, as most foraging time is used to chew and digest, rather than removing plant tissue from the sward [6]. Among items of similar quality, intake rate itself is also an important determinant of choice, as shown for both sheep [6,7] and goats [8]. Intake rate maximization can explain preferences for tall swards [5,9].

Preference and the resulting diet are also expected to vary according to traits of the individual. Energy requirements/intake scales allometrically, while rumen size scale isometrically with body size [10]. It is therefore expected that larger ruminants can persist on a lower quality diet, known as the Jarman-Bell principle [11,12]. Indeed, one of the most important hypothesis of sexual segregation of males and females in sexually size-dimorphic ungulates is based on this principle [13–15]; review in [16,17]. Age classes of different sizes are therefore also expected to differ in their diet. However, recent studies point to a more complex mechanistic explanation of digestive physiology than provided by the Jarman-Bell principle [18]. Other factors may yield further dietary differences depending on age, after controlling for size differences, such as learning [19]. Proximate mechanisms for learning include postingestive feedback [20,21]. Tooth wear may also cause animals change diet as they age, if they become poorer in mastication efficiency [22].

It is well known that diet is affected by environmental conditions having a marked effect on forage availability. At high population density, large herbivores eat a broader diet as they also include dietary items of lower quality [4,23,24]. Diet composition is also affected by prevailing weather conditions, for example depending on snow depth during winter [25], and it varies also among summers depending on vegetation development [23]. The relative importance of environmental variation (population density, climate) driving availability relative to individual traits (age, body mass, experience) affecting preference has not been examined in the same study. Further, it has never been tested how experience animals gain in one environment may be reflected if moved to another except at short time scales [26]. For example, if animals have been foraging in a high density environment, will this affect their future diet if moved to low density?

We here analyse a 10-year dataset of diet composition based on microhistological analysis from 412 individual domestic sheep (*Ovis aries*) within a fully replicated, landscape scale experiment with high and low sheep density in an alpine ecosystem in Norway. We compare the effect of the individual traits age, sex, body mass, litter size with the environmental variables current density, previous density, and variables used as proxy for vegetation development; year and date for the proportional intake of forage. We contrast the proportional intake of herbs (high quality/low availability) and *Avenella flexuosa* (lower quality/high availability), as a high intake of herbs and a low intake of *A*. *flexuosa* in the diet is known to yield higher dietary quality [27].

## Materials and Methods

### Ethics statements

Our study adheres to the “Guidelines for the Use of Animals in Research”, and to the legal requirements of Norway where the work has been carried out. The field studies were carried out on private land with written agreement with the landowner. The activities involves ordinary husbandry practices being well controlled in Norway and requiring no extra permit. The field studies did not involve endangered or protected species.

### Study area and experimental design

The study was situated in Hol municipality, Buskerud county, Norway (60°40´N, 7°55´E) in the lower alpine zones above the forest from 1050 m to 1320 m a.s.l.. Vegetation is dominated by low shrubs with scattered grass-dominated meadows providing the most important feeding areas for sheep [28]. A detailed account of the vegetation is given elsewhere [29,30]. A fenced experimental enclosure split in nine sub-enclosures covering 2.7 km^2^ in total was established in 2001. The treatments high density (80 sheep per km^2^), low density (25 sheep per km^2^) or control (no sheep) were replicated 3 times randomized within three blocks. The grazing season was from late June to late August or early September for each year 2002–2011, with an average of 70 days of grazing. All the sheep were of the same breed (“Norsk Kvit Sau”), and ewes had lambs (1–3) and were lactating at time of release. Further details on the annual specific number of sheep in each enclosure and dates of grazing are given elsewhere [30,31].

### Data on sheep diet composition

All sheep were individually marked and followed for the entire grazing season by aid of direct observation [23,24,32]. During these observations, faeces from known individuals were sampled. The faeces were put in plastic bags in the field and frozen. The samples were stratified according to densities (high vs. low), age classes (ewe vs. lamb) and 3 periods (early, middle and late grazing season) to get a more balanced dataset. We obtained 861 samples from 412 individual sheep for all years 2002–2011 (Table 1). Microhistological analyses [33–35] were performed following a standard procedure boiling 1 ml of faeces in 4 ml of 65% concentrated nitric acid [23,24,36]. Plant fragments were identified to species level whenever possible, otherwise family names were determined. Two parallel sub-samples were processed independently (343 samples) for 2002–2006, but not for years 2007–2011. The mean number of faeces samples analyzed per individual sheep was 2.09 (±1.89 SD), and 2.92 (±2.49 SD) if including the two parallel subsamples.

**Table 1:** Sample size.

	2002	2003	2004	2005	2006	2007	2008	2009	2010	2011	Sum
**Ewes**											
**high**	30	38	28	34	44	26	32	33	24	33	322
**low**	26	30	26	32	44	28	26	21	18	14	265
**Lambs**											
**high**	28	28	38	42	38	21	26	33	27	33	314
**low**	32	24	34	46	44	23	26	23	23	28	303
**Sum**	116	120	126	154	170	98	110	110	92	108	1204

Sample sizes of faeces used in microhistological analysis of diet from sheep in Norway. Samples are broken down to age classes (ewe, lambs), population density (high, low) and years. Note that for years 2002–2006, sample size includes two parallels of the same faeces.

By chance, some individuals switched between treatments from one year to the next. Most samples derive from individuals being in their first year in the experiment (n = 945). There were also samples from 2^nd^ year if remaining at low (n = 33) and high (n = 112) density, and from sheep changing from high to low (n = 70) and low to high (n = 44) density between years. Among these, 97 samples come from 16 individuals that started as lambs and were used in further years as they were ewes.

### Statistical analyses

Based on previous analysis of a subset of the data [23], we focused our analysis on the proportion of the two main dietary components: herbs as a group represent high quality forage, while the grass *A*. *flexuosa* providing the bulk forage [23,24,36]. As response variables were proportions, we arcsinsqrt-transformed them prior to analysis. Analyses were performed using linear effect mixed-models with the “lmer” function in library “lme4” in R. We used model selection with Akaike Information Criterion (AIC) to find the most parsimonious model [37]. In all models, random terms modeled as random intercepts were “individual ID” and “subenclosure”.

Individual traits. As covariates differ between lambs and ewes, we ran analyses for 1) all data and a limited set of covariates, 2) lambs only, and 3) ewes only, with the same baseline model including population density, year as factor (2002–2011) and Julian date. 1) In the full model, covariates were age and (ln) body mass. We tried several ways to model age; age, age^2^, age with smoothing spline (using library “splines”), age categorical (ewe vs. lamb) and fully age categorical (years 0–7). As lambs almost double in mass over the summer, we also calculated an adjusted body mass, based on calculated daily growth rates ([autumn mass-spring mass]/grazing days) for each individual. Adjusted body mass is hence estimated to the date of faecal sampling. If values were missing, we replaced with spring mass for ewes as they have fairly stable mass (unpublished data), while missing values were removed for lambs. 2) For lambs only, covariates were litter size (1–3 levels), sex, (ln) body mass (spring or adjusted), age of ewe, and (ln) mass of ewe. 3) For ewes only, potential predictor variables were age, (ln) body mass and litter size.

Environmental variables. The initial model for selecting environmental variables was the best model on individual traits.

## Results

### Individual traits

The best model identified (ln) body mass as the best predictor for herbs in the diet, being ranked before age category with two levels (ewe/lamb) and far above other models with age, age^2^, age as spline, or age as fully categorical (7 levels) (Table 2). Adjusted mass (to the date of faecal collection) did not further improve model fit relative to spring mass (AIC[mass] = -1814, AIC[adj.mass] = -1799).

**Table 2:** Model selection.

age	age2	Spline (age)	age categorical (ewe vs lamb)	age categorical (0–7)	Ln (body mass)	density	year categorical	Julian date	AIC	ΔAIC
Herbs						1	1	1	-1779	52
1						1	1	1	-1808	23
1	1					1	1	1	-1817	14
		1				1	1	1	-1822	9
			1			1	1	1	-1825	6
				1		1	1	1	-1794	37
					1	1	1	1	-1831	0
*A*. *flexuosa*						1	1	1	-1448	73
1						1	1	1	-1499	22
1	1					1	1	1	-1500	21
		1				1	1	1	-1505	16
			1			1	1	1	-1521	0
				1		1	1	1	-1490	31
					1	1	1	1	-1517	4

Model selection for proportion of herbs and *A*. *flexuosa* in the diet of sheep including all ages 0–7 yrs. ID and sub-enclosure were random terms in all models.

The best model for proportion of *A*. *flexuosa* in the sheep diet included age category with two levels (ewe/lamb), being ranked markedly before (ln) body mass, and far above other models with age, age^2^, age as spline, or age as fully categorical (7 levels) (Table 2). This result was robust when using body mass adjusted to the date of faecal collection (AIC[age cat] = -1497; vs. AIC[adj.mass] = -1486).

**Fig 1:**
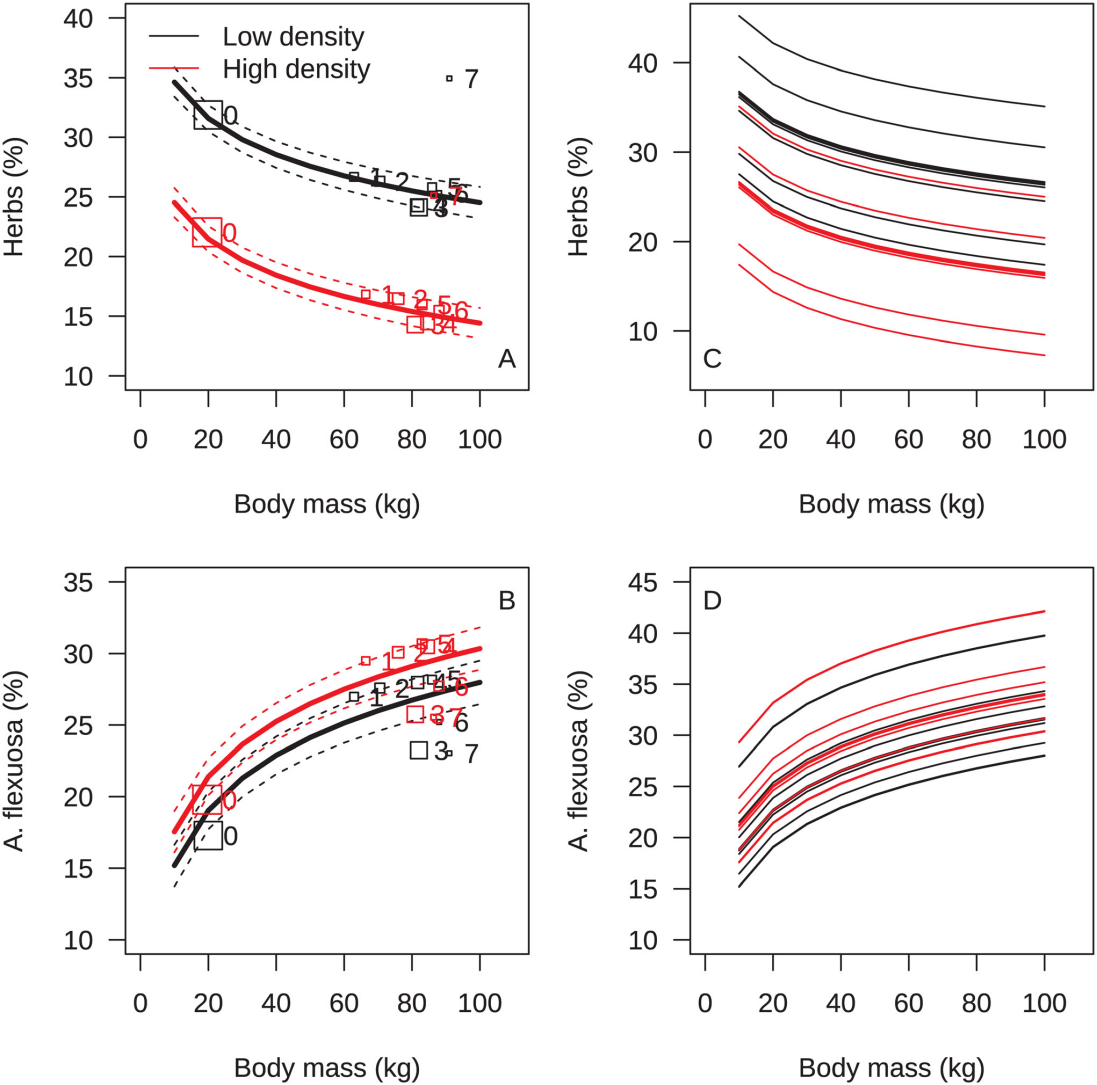
Diet. The dietary proportion of (A) herbs and (B) *Avenella flexuosa* as a function of body mass with average values for each age class (number 0–7) superimposed (for year 2008 and Julian date = 200). For herbs, the best model included (ln) body mass, while for *Avenella flexuosa*, the best model included age classes ewe vs. lamb. The squares are proportional to sqrt(sample size). Figures C and D are lines for each year 2002–2011 for high and low density.

For models with lambs only, the baseline model for both herbs and *A*. *flexuosa* including only environmental variables (density, year, date) outcompeted more complex models with sex, body mass, litter size, age and body mass of ewe (Table 3). For models with ewes only, the baseline model including only environmental variables (density, year, date) outcompeted more complex models with age, age^2^, age as spline, or age as fully categorical (6 levels), or litter size, while the model including body mass was competitive for *A*. *flexuosa* (Table 4). Thus, both age category (ewe/lamb) and body mass had some merit in predicting proportional intake of the most common dietary items (Fig 1A and 1B).

**Table 3:** Model selection 2.

	sex	Ln (body mass)	Ln (adj. body mass)	age of ewe	spline(age)	Ln (body mass of ewe)	litter size	density	year categorical	Juliandate	ΔAIC
Herbs								1	1	1	0
	1							1	1	1	5.9
		1						1	1	1	2.7
			1					1	1	1	5.9
				1				1	1	1	8.9
					1			1	1	1	12.4
						1		1	1	1	6.1
							1	1	1	1	8.6
*A*. *flexuosa*								1	1	1	0
	1							1	1	1	8.2
		1						1	1	1	7.2
			1					1	1	1	4.8
				1				1	1	1	10.6
					1			1	1	1	14.1
						1		1	1	1	6
							1	1	1	1	14.7

Model selection for proportion of herbs and *A*. *flexuosa* in the diet of sheep lambs. Note that due to missing values, sample size differs slightly between models. Therefore only ΔAIC is reported, and always comparisons were made on the same sample. ID and sub-enclosure was random terms in all models.

**Table 4:** Model selection 3.

	age	Age 2	spline(age)	age categorical (1–7)	Ln (body mass)	litter size	density	year categorical	Juliandate	AIC	ΔAIC
Herbs							1	1	1	-866.9	0
	1						1	1	1	-855.9	11
	1	1					1	1	1	-846.6	20.3
			1				1	1	1	-858.4	8.5
				1			1	1	1	-836.7	30.2
					1		1	1	1	-860.9	6
						1	1	1	1	-849.6	17.3
*A*. *flexuosa*							1	1	1	-672.2	0
	1						1	1	1	-662.2	10
	1	1					1	1	1	-651.4	20.8
			1				1	1	1	-659.8	12.4
				1			1	1	1	-634.4	37.8
					1		1	1	1	-671.1	1.1
						1	1	1	1	-657.5	14.7

Model selection for proportion of herbs and *A*. *flexuosa* in the diet of ewes. ID and sub-enclosure was random terms in all models.

### Environmental variation

Population density markedly reduced proportional intake of herbs (Table 5). The proportional intake of herbs in the diet declined as the grazing season progressed, and was replaced by a higher proportion of *A*. *flexuosa*. There was a marked annual variation in proportional intake of herbs (Fig 1C). Overall, proportional intake of *A*. *flexuosa* increased at high density and showed some annual variation, but less marked than for herbs (Fig 1D). Part of this was due to interaction between density and the annual variation (as previously reported in [23] for a shorter subset of the data), however, the model including the interaction between density and year was not favored for this longer period (AIC [without interaction] = -1831; AIC[with interaction] = -1821).

**Table 5:** Estimates from the best models.

Parameter	Estimate	Std. Error	Lower limit	Upper limit
**Herbs**				
Intercept	1.162	0.050	1.061	1.262
Density (low vs. high)	0.121	0.034	0.052	0.189
I(juliandate)	-0.003	0.000	-0.003	-0.002
log(springmass)	-0.054	0.007	-0.067	-0.040
Year (2003 vs.2002)	0.096	0.019	0.058	0.135
Year (2004 vs.2002)	-0.121	0.019	-0.160	-0.082
Year (2005 vs.2002)	0.041	0.019	0.002	0.079
Year (2006 vs.2002)	-0.009	0.019	-0.047	0.029
Year (2007 vs.2002)	-0.009	0.020	-0.049	0.030
Year (2008 vs.2002)	-0.022	0.019	-0.060	0.016
Year (2009 vs.2002)	0.000	0.019	-0.038	0.037
Year (2010 vs.2002)	-0.014	0.020	-0.053	0.025
Year (2011 vs.2002)	-0.093	0.019	-0.132	-0.054
***A*. *flexuosa***				
Intercept	-0.264	0.047	-0.358	-0.171
Density (low vs. high)	-0.026	0.032	-0.089	0.038
I(juliandate)	0.004	0.000	0.004	0.005
Age cat (lamb vs. ewe)	-0.100	0.010	-0.121	-0.079
Year (2003 vs.2002)	-0.012	0.021	-0.054	0.029
Year (2004 vs.2002)	0.070	0.021	0.029	0.112
Year (2005 vs.2002)	-0.027	0.020	-0.068	0.014
Year (2006 vs.2002)	-0.055	0.020	-0.095	-0.014
Year (2007 vs.2002)	-0.025	0.021	-0.068	0.017
Year (2008 vs.2002)	-0.058	0.020	-0.099	-0.017
Year (2009 vs.2002)	-0.018	0.020	-0.058	0.022
Year (2010 vs.2002)	0.016	0.021	-0.026	0.058
Year (2011 vs.2002)	0.078	0.021	0.037	0.119

Estimates from the best models (Table 2) explaining proportional intake of herbs and *A*. *flexuosa* in sheep 2002–2011 in an alpine ecosystem, Norway.

## Discussion

There is a renewed interest in large herbivore diet composition due to issues related to biodiversity preservation [38]. Dry matter intake rates of ewes are estimated in the range 2–3 kg per day [39,40]. Clearly, the composition of what they eat affects both performance of the ruminants themselves and the ecosystem function [1]. In our experiment for example, sheep selection for tall herb species was a predictor of which herb species declined or increased in abundance over time [41]. Our analyses of 10 years of data on diet composition of sheep in an alpine ecosystem document strong effects of the immediate environment related to population density and proxies for vegetation development (year, date) and hence forage availability, while their individual traits linked to life history was less important. The only important individual trait was the body mass and/or the age difference between lambs and ewes explaining differences in preference of herbs and the bulk forage grass *A*. *flexuosa*, respectively.

### Individual traits

Herbs as a group and *A*. *flexuosa* constitutes the major part of the sheep diet (Fig 1). It is clear that large herbivore diet is always a mixture of items, termed partial preferences. Why such partial dietary preferences remain is debated. Some argue partial preference is due to discrimination error between alternatives of similar intake rate [8]. However, sheep tend to balance diet to get both protein, essential nutrients and energy [42], and therefore change diet depending on previous diet’s nutritional content [43]. Protein content of herbs in our area is somewhat higher than for *A*. *flexuosa*, though *A*. *flexuosa* retain a relatively high N-content at the end of the grazing season in particular if grazed at high levels [27]. However, *A*. *flexuosa* is very much more abundant than the herbs [30]. Herbs thus represent high quality and low availability and the reverse for *A*. *flexuosa*. Therefore, the pattern we observe relative to density, year and date effects of the relative balance of herbs vs. *A*. *flexuosa* is to a large extent likely driven by differences in availability. However, individual variation after accounting for such environmental variation can be inferred as differences in preference.

Consistent with earlier analyses on shorter time series, diet differed markedly between lambs and ewes [23,24]. For *A*. *flexuosa*, animal age category was a better model of diet composition than (ln) body mass. If learning plays a role, diet composition due to age class may be more than a matter of body mass differences. However, for herbs we found the reverse, the better predictor of proportional intake was (ln) body mass rather than sheep age category. For ewes only, the models including body mass were better than several ways to model age (≥1), but none was better than the baseline model with only environmental factors. Age category and body mass are obviously highly linked over the full range of age from lambs to ewes. Our analysis suggested that due to this strong link between sheep age category and body mass, it was somewhat random which of the two traits body mass or age category (lamb/ewe) was identified in the best model (Fig 1). In another study, lactating ewes consumed more roughage than non-lactating ewes [39], and for lactating cows had higher overall intake rates [44]. In our case, all ewes were lactating, but such factors may add to the difference between ewes and lambs beyond those of body mass. The pattern of more herbs (high quality, low availability) for small individuals/lambs relative to larger ewes is therefore consistent with predictions expected from general theory on size specific diet choice [10].

We have for this experiment found that growth of all lambs decreases with increased litter size, and that larger lambs in spring grow relatively more body mass [45]. However, diet composition of lambs was unaffected by lamb sex, body mass, litter size, age or mass of mother. We can thus conclude that most variation in growth of lambs after controlling for environmental variation does not arise due to difference in proportional intake of different forages.

### Current and previous environmental effects

The effect of current environmental variation related to animal population density, annual variation and season on diet composition was marked (see also [23]). Clearly, diet choice is to a large extent driven by environmentally driven variation in availability, and year and date are proxies for vegetation development. Lagged environmental effects are central in population ecology. Early conditions may affect life time performance of ungulates and thus create cohort effects (review in [46]). In cyclic populations of small mammals, performance of individuals differ for the same population density depending on whether the population is increasing or decreasing [47,48], and it has been discussed whether this variation arises due to changes in the environment or within the individual. Experimentation with moving *Microtus* voles between areas in different phases of population cycles determined that voles respond to the immediate environment [49,50]. Foraging in sheep and other ungulates is markedly affected by learning and thus previous experience [21,51]. The sequence of dietary items may play a role in intake of sheep if they contain different chemical substances [52]. However, we failed to find any effect of whether a sheep had grazed the previous year in a different density treatment. It is possible that stronger effects might be found if comparing only the transition from the environment experienced as lamb, since clearly most learning likely happened during that stage [26]. We failed to find evidence for this, but we only had 16 lambs included in the data, so power was likely somewhat low if such effects are subtle.

## Conclusion

Our study highlights that diet composition of a ruminant, domestic sheep, arise mainly as a function of lamb and ewes age and/or body mass differences, and the immediate environment in terms of population density and proxies for vegetation development (year and date). Predicting forage offtake by herbivores is central to management [40], and our study provides such baseline information for management at annual and seasonal scales and depending on demographic composition of a herd.

## Supporting Information

S1 File Data used to model sheep diet. (PDF)
