# Supplementary material for: The Role of Individual Traits and Environmental Factors for Diet Composition of Sheep
Source: PLoS One. 2016 Jan 5;11(1):e0146217. doi: 10.1371/journal.pone.0146217 (PMC4701509; doi:10.1371/journal.pone.0146217)
Supplement: S1 File — (PDF) [file pone.0146217.s001.pdf]

density	sex	agecat	year	adjMass	littersize	juliandate	Aflexuosa	Herbs
low	female	lamb	2002	16	asingleton	199	23.4	14.6
low	female	lamb	2002	16	asingleton	199	23.7	16.8
low	female	lamb	2002	17	asingleton	206	30.1	5
low	female	lamb	2002	17	asingleton	206	30	7.4
high	female	lamb	2002	20	triplet	199	40.1	30.7
high	female	lamb	2002	20	triplet	199	42.7	32.3
high	female	lamb	2002	21	triplet	185	22	16.4
high	female	lamb	2002	21	triplet	185	23.4	15.3
low	male	lamb	2002	21	twin	190	8.2	22.9
low	male	lamb	2002	21	twin	190	9.4	21.6
low	male	lamb	2002	22	triplet	178	15.2	23.7
low	male	lamb	2002	22	triplet	178	16.7	25.3
high	male	lamb	2002	23	triplet	216	30	31.4
high	male	lamb	2002	23	triplet	216	30.8	28.9
low	male	lamb	2002	23	twin	194	14.1	41.1
low	male	lamb	2002	23	twin	194	13.9	37.2
low	male	lamb	2002	24	asingleton	187	26.9	16.3
low	male	lamb	2002	24	asingleton	187	23.2	19.8
high	female	lamb	2002	25	triplet	207	32.3	15.9
high	female	lamb	2002	25	triplet	207	31.5	20.1
high	male	lamb	2002	27	triplet	203	37.3	10.4
high	male	lamb	2002	27	triplet	203	34.6	13.8
high	male	lamb	2002	29	asingleton	180	16.8	18.6
high	male	lamb	2002	29	asingleton	180	15.6	14.8
high	male	lamb	2002	29	twin	203	17.5	48.8
high	male	lamb	2002	29	twin	203	14.2	47.2
high	female	lamb	2002	29	twin	218	26.3	14.8
high	female	lamb	2002	29	twin	218	30.4	14.6
low	female	lamb	2002	29	twin	200	34.1	24.4
low	female	lamb	2002	29	twin	200	37.8	28.3
high	female	lamb	2002	29	twin	202	11.1	10.6
high	female	lamb	2002	29	twin	202	10.7	12.2
high	female	lamb	2002	30	twin	210	29.1	11.4
high	female	lamb	2002	30	twin	210	26.5	14.2
low	male	lamb	2002	31	asingleton	203	20.8	11.6
low	male	lamb	2002	31	asingleton	203	22.5	13.8
high	male	lamb	2002	33	triplet	238	61.4	17.1
high	male	lamb	2002	33	triplet	238	59.4	17.7
low	male	lamb	2002	34	asingleton	202	8.9	39.2
low	male	lamb	2002	34	asingleton	202	10.6	41.6
low	female	lamb	2002	34	twin	195	19.8	27.6
low	female	lamb	2002	34	twin	195	18.1	30.3
low	female	lamb	2002	37	triplet	240	36.9	42.2
low	female	lamb	2002	37	triplet	240	34	46
low	male	lamb	2002	37	twin	241	27.8	29.2
low	male	lamb	2002	37	twin	241	25.2	32.1
high	male	lamb	2002	38	twin	240	64.9	19.8
high	male	lamb	2002	38	twin	240	63	23.5
high	female	lamb	2002	38	triplet	240	40.4	15.9
high	female	lamb	2002	38	triplet	240	41.4	17.1
high	male	lamb	2002	44	twin	211	40	11.4
high	male	lamb	2002	44	twin	211	38.9	10.3
low	male	lamb	2002	47	asingleton	241	9.9	5.8
low	male	lamb	2002	47	asingleton	241	10.9	6.1
low	male	lamb	2002	48	twin	204	32.8	26.1

low	male	lamb	2002	48	twin	204	36	28.5
low	NA	ewe	2002	50	asingleton	233	37.5	15.5
low	NA	ewe	2002	50	asingleton	233	34.4	18.8
low	male	lamb	2002	52	twin	217	21.3	18.3
low	male	lamb	2002	52	twin	217	22	16.5
low	NA	ewe	2002	55	asingleton	180	18.6	27
low	NA	ewe	2002	55	asingleton	180	15.4	29.5
low	NA	ewe	2002	59	asingleton	187	36.3	9.3
low	NA	ewe	2002	59	asingleton	187	33	10.3
low	NA	ewe	2002	63	asingleton	212	22.4	10.4
low	NA	ewe	2002	63	asingleton	212	23.5	10.7
low	NA	ewe	2002	69	twin	184	33.8	29.2
low	NA	ewe	2002	69	twin	184	33.6	30.9
low	NA	ewe	2002	71	twin	241	37	20.5
low	NA	ewe	2002	71	twin	241	40.4	17.4
high	NA	ewe	2002	73	asingleton	202	33	20.1
high	NA	ewe	2002	73	asingleton	202	30	23.1
high	NA	ewe	2002	73	twin	199	38.4	32.7
high	NA	ewe	2002	73	twin	199	34.7	33.9
high	female	ewe	2002	76	twin	184	34.5	20.4
high	female	ewe	2002	76	twin	184	31.7	20.2
high	NA	ewe	2002	78	twin	233	49.5	9.2
high	NA	ewe	2002	78	twin	233	51.1	10.3
high	NA	ewe	2002	81	asingleton	178	29.5	14.3
high	NA	ewe	2002	81	asingleton	178	26.3	18.2
low	NA	ewe	2002	82	asingleton	178	39.1	32.2
low	NA	ewe	2002	82	asingleton	178	35.6	29.5
high	NA	ewe	2002	84	twin	187	45.6	26.6
high	NA	ewe	2002	84	twin	187	43.2	28.1
high	NA	ewe	2002	84	asingleton	218	38.1	11.4
high	NA	ewe	2002	84	asingleton	218	39.8	14.6
low	NA	ewe	2002	84	twin	200	31	26.6
low	NA	ewe	2002	84	twin	200	31.3	26.7
low	NA	ewe	2002	88	twin	233	15.1	38.7
low	NA	ewe	2002	88	twin	233	13.9	40.1
high	NA	ewe	2002	88	triplet	213	39.9	22.7
high	NA	ewe	2002	88	triplet	213	43.5	21.1
high	NA	ewe	2002	88	twin	200	18.3	25.6
high	NA	ewe	2002	88	twin	200	15.9	24.3
high	NA	ewe	2002	88	triplet	184	36.3	17.1
high	NA	ewe	2002	88	triplet	184	34.8	22
low	NA	ewe	2002	90	asingleton	188	34.7	26.2
low	NA	ewe	2002	90	asingleton	188	30	30.1
low	NA	ewe	2002	92	asingleton	234	21.3	21.7
low	NA	ewe	2002	92	asingleton	234	25.6	19.5
high	NA	ewe	2002	92	triplet	240	55	11.3
high	NA	ewe	2002	92	triplet	240	57.7	12.1
low	NA	ewe	2002	92	asingleton	241	26.3	25.3
low	NA	ewe	2002	92	asingleton	241	32.6	24.6
low	NA	ewe	2002	92	triplet	214	32.2	38.2
low	NA	ewe	2002	92	triplet	214	30.6	35.4
high	NA	ewe	2002	96	triplet	178	33.9	5.9
high	NA	ewe	2002	96	triplet	178	29.4	11.4
high	female	ewe	2002	98	asingleton	207	49.3	17.4
high	female	ewe	2002	98	asingleton	207	48.2	14.7
high	female	lamb	2003	21	asingleton	200	6.6	46.9

high	female	lamb	2003	21	asingleton	200	6.6	45.1
high	NA	lamb	2003	22	twin	191	6.1	68.6
high	NA	lamb	2003	22	twin	191	5.3	66.5
low	female	lamb	2003	22	asingleton	204	24.2	31.2
low	female	lamb	2003	22	asingleton	204	26	34.5
low	female	lamb	2003	22	twin	183	32.1	28.6
low	female	lamb	2003	22	twin	183	32.4	31.9
low	male	lamb	2003	23	triplet	187	24.3	49.2
low	male	lamb	2003	23	triplet	187	26.3	49.6
low	male	lamb	2003	24	triplet	191	10.7	53.9
low	male	lamb	2003	24	triplet	191	11.1	53.9
high	male	lamb	2003	25	twin	184	18.9	31.3
high	male	lamb	2003	25	twin	184	19.5	31.7
high	female	lamb	2003	25	twin	202	19.1	41.4
high	female	lamb	2003	25	twin	202	17	43.6
low	female	lamb	2003	26	asingleton	188	19.5	22.6
low	female	lamb	2003	26	asingleton	188	20.4	22.7
low	female	lamb	2003	27	twin	209	22.7	28.9
low	female	lamb	2003	27	twin	209	22.8	33.3
low	female	lamb	2003	27	twin	186	30.2	21.9
low	female	lamb	2003	27	twin	186	31.7	26.8
high	male	lamb	2003	27	asingleton	202	27.2	36.1
high	male	lamb	2003	27	asingleton	202	25.3	38.5
low	male	lamb	2003	29	asingleton	201	23.1	35.6
low	male	lamb	2003	29	asingleton	201	26.3	33.3
low	male	lamb	2003	29	asingleton	187	24.6	22.6
low	male	lamb	2003	29	asingleton	187	22.5	26
high	female	lamb	2003	30	twin	202	52.6	18.7
high	female	lamb	2003	30	twin	202	48.6	19.4
high	male	lamb	2003	30	asingleton	210	28.9	29.3
high	male	lamb	2003	30	asingleton	210	27.2	31.1
low	male	lamb	2003	30	asingleton	189	39.5	29.8
low	male	lamb	2003	30	asingleton	189	36.2	30.7
low	female	lamb	2003	35	asingleton	211	20.2	29.3
low	female	lamb	2003	35	asingleton	211	24.4	28.4
high	female	lamb	2003	35	twin	231	23	31.1
high	female	lamb	2003	35	twin	231	19.9	33.8
high	female	lamb	2003	36	twin	209	33.3	47.7
high	female	lamb	2003	36	twin	209	37.1	50.2
high	male	lamb	2003	37	twin	209	16.2	27.2
high	male	lamb	2003	37	twin	209	17	27
high	female	lamb	2003	37	twin	231	26	30.7
high	female	lamb	2003	37	twin	231	28.8	30.9
low	male	lamb	2003	42	triplet	235	32	25.6
low	male	lamb	2003	42	triplet	235	34.9	25.2
high	female	lamb	2003	43	twin	230	24.7	54
high	female	lamb	2003	43	twin	230	26.2	49
low	NA	ewe	2003	63	asingleton	183	18.1	41.7
low	NA	ewe	2003	63	asingleton	183	19.5	42.9
low	NA	ewe	2003	63	asingleton	185	27.6	29.5
low	NA	ewe	2003	63	asingleton	185	27.5	29.3
high	NA	ewe	2003	74	twin	233	47.1	16.3
high	NA	ewe	2003	74	twin	233	47.7	19.6
high	NA	ewe	2003	74	twin	233	55.1	13.6
high	NA	ewe	2003	74	twin	233	55.1	15
high	NA	ewe	2003	75	twin	183	24.9	16.6

high	NA	ewe	2003	75 twin	183	22.9	15.6	
high	NA	ewe	2003	76 triplet	183	15.5	34.8	
high	NA	ewe	2003	76 triplet	183	15.8	37.1	
high	NA	ewe	2003	77 triplet	202	31.1	28	
high	NA	ewe	2003	77 triplet	202	31.4	28	
high	NA	ewe	2003	79 twin	230	60.7	19.4	
high	NA	ewe	2003	79 twin	230	61.6	17	
high	female	ewe	2003	80 twin	199	32.2	20.9	
high	female	ewe	2003	80 twin	199	33.8	18.6	
low	NA	ewe	2003	82 asingleton	211	38	16.7	
low	NA	ewe	2003	82 asingleton	211	36.3	16.7	
high	NA	ewe	2003	83 twin	182	35.6	41.5	
high	NA	ewe	2003	83 twin	182	34.7	42.9	
low	NA	ewe	2003	83 asingleton	204	30.6	24.4	
low	NA	ewe	2003	83 asingleton	204	27.8	27.4	
low	NA	ewe	2003	84 asingleton	186	24.9	29.4	
low	NA	ewe	2003	84 asingleton	186	27.8	30.2	
low	NA	ewe	2003	84 asingleton	183	39	26.4	
low	NA	ewe	2003	84 asingleton	183	35.4	27.1	
high	NA	ewe	2003	84 twin	235	60	8.2	
high	NA	ewe	2003	84 twin	235	57.3	5.8	
high	NA	ewe	2003	85 twin	231	32.6	18.3	
high	NA	ewe	2003	85 twin	231	37	22.2	
low	NA	ewe	2003	85 asingleton	191	28.8	37	
low	NA	ewe	2003	85 asingleton	191	28.3	35.8	
low	NA	ewe	2003	85 asingleton	189	21	56.9	
low	NA	ewe	2003	85 asingleton	189	19.1	55.9	
high	female	ewe	2003	85 twin	185	15.6	62.7	
high	female	ewe	2003	85 twin	185	17.2	59.9	
high	female	ewe	2003	86 twin	233	50	27.6	
high	female	ewe	2003	86 twin	233	49.3	22.5	
low	NA	ewe	2003	86 twin	235	42.1	32.1	
low	NA	ewe	2003	86 twin	235	46.2	33.3	
high	NA	ewe	2003	90 triplet	192	41.5	35.5	
high	NA	ewe	2003	90 triplet	192	39	37.1	
low	female	ewe	2003	91 asingleton	181	46.6	25	
low	female	ewe	2003	91 asingleton	181	45	27	
low	female	ewe	2003	91 asingleton	187	26.8	37	
low	female	ewe	2003	91 asingleton	187	21.8	38.3	
low	female	ewe	2003	91 asingleton	189	18	41.7	
low	female	ewe	2003	91 asingleton	189	18.9	45	
low	female	ewe	2003	92 asingleton	204	23.2	37.2	
low	female	ewe	2003	92 asingleton	204	25.7	37.6	
high	NA	ewe	2003	92 asingleton	236	41.4	15.3	
high	NA	ewe	2003	92 asingleton	236	38.9	18.8	
high	NA	ewe	2003	94 asingleton	199	26.5	49.5	
high	NA	ewe	2003	94 asingleton	199	20.6	45	
high	NA	ewe	2003	99 asingleton	182	28.2	45.4	
high	NA	ewe	2003	99 asingleton	182	27.8	44	
high	NA	ewe	2003	100 asingleton	236	67.4	12.5	
high	NA	ewe	2003	100 asingleton	236	70.3	13.7	
low	NA	ewe	2003	100 twin	182	21.4	23.2	
low	NA	ewe	2003	100 twin	182	17.8	25.5	
low	NA	ewe	2003	100 twin	189	71.7	5.4	
low	NA	ewe	2003	100 twin	189	68.6	5.3	
high	NA	ewe	2003	100 asingleton	233	54.1	9.5	

high	NA	ewe	2003	100	asingleton	233	54.7	10.8
high	male	ewe	2003	101	asingleton	233	48.3	26.6
high	male	ewe	2003	101	asingleton	233	44.3	24
low	male	lamb	2004	14	triplet	185	40.4	12.8
low	male	lamb	2004	14	triplet	185	39.2	15.8
high	female	lamb	2004	16	twin	184	43.8	13.5
high	female	lamb	2004	16	twin	184	42.7	15.1
low	male	lamb	2004	22	twin	185	41.5	13
low	male	lamb	2004	22	twin	185	38.2	16.8
low	female	lamb	2004	22	triplet	230	36.9	5.8
low	female	lamb	2004	22	triplet	230	33.8	7
low	male	lamb	2004	23	twin	189	40.1	19.4
low	male	lamb	2004	23	twin	189	42.8	18.5
low	female	lamb	2004	23	triplet	182	24.9	13.2
low	female	lamb	2004	23	triplet	182	21.3	17.1
high	male	lamb	2004	23	triplet	193	29.1	31.6
high	male	lamb	2004	23	triplet	193	27.3	34.1
high	male	lamb	2004	27	twin	182	30	19.6
high	male	lamb	2004	27	twin	182	27.7	22.1
low	female	lamb	2004	27	asingleton	193	31.2	21.2
low	female	lamb	2004	27	asingleton	193	31	24.4
low	female	lamb	2004	28	triplet	196	17.5	25.7
low	female	lamb	2004	28	triplet	196	18.9	24.5
high	female	lamb	2004	29	twin	182	27.2	21.1
high	female	lamb	2004	29	twin	182	25.8	22.9
high	male	lamb	2004	29	twin	229	34.9	11.8
high	male	lamb	2004	29	twin	229	36.9	10.7
low	male	lamb	2004	29	triplet	234	57.6	12.1
low	male	lamb	2004	29	triplet	234	57.2	11.8
high	NA	lamb	2004	29	twin	194	22.6	19.6
high	NA	lamb	2004	29	twin	194	21.4	17.9
low	male	lamb	2004	30	twin	195	46.8	13.5
low	male	lamb	2004	30	twin	195	42.5	16.2
high	male	lamb	2004	31	twin	182	16.2	35
high	male	lamb	2004	31	twin	182	14.2	37.7
low	male	lamb	2004	32	twin	215	44.8	18.9
low	male	lamb	2004	32	twin	215	44.2	19.7
high	female	lamb	2004	33	twin	195	11.6	26.7
high	female	lamb	2004	33	twin	195	14.3	27.3
high	female	lamb	2004	33	asingleton	216	33.8	14.3
high	female	lamb	2004	33	asingleton	216	33.6	16.6
low	NA	lamb	2004	35	asingleton	184	18.7	22.4
low	NA	lamb	2004	35	asingleton	184	16.5	22.9
low	male	lamb	2004	35	triplet	241	50.2	10.8
low	male	lamb	2004	35	triplet	241	51.9	8.6
high	male	lamb	2004	35	triplet	218	23.6	12.3
high	male	lamb	2004	35	triplet	218	25.3	13.8
low	male	lamb	2004	35	twin	229	47.2	15.3
low	male	lamb	2004	35	twin	229	48.7	14.9
low	female	lamb	2004	36	triplet	220	17.6	25.1
low	female	lamb	2004	36	triplet	220	17.9	25
high	female	lamb	2004	36	twin	234	31.1	6.7
high	female	lamb	2004	36	twin	234	30.3	3.8
high	female	lamb	2004	36	twin	241	55.1	9.7
high	female	lamb	2004	36	twin	241	53.3	9.2
high	female	lamb	2004	36	asingleton	228	36.9	9

high	female	lamb	2004	36	asingleton	228	38.6	10.5
high	female	lamb	2004	37	triplet	239	28.6	12
high	female	lamb	2004	37	triplet	239	30.3	9.7
low	female	lamb	2004	39	twin	241	58.1	14.8
low	female	lamb	2004	39	twin	241	59.8	12.8
high	male	lamb	2004	40	triplet	239	53.1	15.9
high	male	lamb	2004	40	triplet	239	53.3	14.8
low	female	lamb	2004	40	triplet	232	35.3	4.8
low	female	lamb	2004	40	triplet	232	34.7	6.9
high	male	lamb	2004	42	asingleton	223	20.7	2.3
high	male	lamb	2004	42	asingleton	223	19.9	4.1
high	male	lamb	2004	45	twin	230	24.5	2
high	male	lamb	2004	45	twin	230	24.7	2.4
high	male	lamb	2004	45	asingleton	234	24.6	10.9
high	male	lamb	2004	45	asingleton	234	27.5	11.2
high	male	lamb	2004	50	twin	218	34	11.9
high	male	lamb	2004	50	twin	218	33.3	13.6
low	NA	lamb	2004	56	asingleton	234	47.6	10.3
low	NA	lamb	2004	56	asingleton	234	45.8	13.1
low	male	lamb	2005	18	triplet	186	19.8	43.6
low	male	lamb	2005	18	triplet	186	21	39.1
low	female	lamb	2005	20	triplet	199	42.1	27.1
low	female	lamb	2005	20	triplet	199	39	27.7
low	female	lamb	2005	21	triplet	202	42	20.4
low	female	lamb	2005	21	triplet	202	39.7	18.7
high	female	lamb	2005	22	twin	194	6.8	25.6
high	female	lamb	2005	22	twin	194	8.4	26
high	female	lamb	2005	22	twin	196	21.2	43.4
high	female	lamb	2005	22	twin	196	23	42.1
low	female	lamb	2005	25	triplet	201	27.7	36.8
low	female	lamb	2005	25	triplet	201	26.5	38
high	female	lamb	2005	25	asingleton	189	12.2	26
high	female	lamb	2005	25	asingleton	189	15.3	24
low	female	lamb	2005	26	triplet	188	22.2	41
low	female	lamb	2005	26	triplet	188	24.2	41
low	male	lamb	2005	27	triplet	215	29	29.5
low	male	lamb	2005	27	triplet	215	28.6	26.8
low	male	lamb	2005	27	asingleton	187	12.9	46.5
low	male	lamb	2005	27	asingleton	187	14.2	47.2
high	male	lamb	2005	28	twin	215	20.7	54.4
high	male	lamb	2005	28	twin	215	21.9	57.2
low	male	lamb	2005	30	twin	215	43	28.1
low	male	lamb	2005	30	twin	215	42.7	31.1
low	female	lamb	2005	30	asingleton	198	8.8	37.9
low	female	lamb	2005	30	asingleton	198	8.5	37.9
high	female	lamb	2005	30	twin	201	9.2	31.3
high	female	lamb	2005	30	twin	201	11.2	31.7
high	female	lamb	2005	30	twin	227	16.4	23.7
high	female	lamb	2005	30	twin	227	17.8	23.4
high	female	lamb	2005	30	twin	228	24.3	26.1
high	female	lamb	2005	30	twin	228	26.7	25.7
high	female	lamb	2005	31	triplet	219	24.3	29.1
high	female	lamb	2005	31	triplet	219	23	27
low	male	lamb	2005	31	triplet	228	27.9	28.3
low	male	lamb	2005	31	triplet	228	30.3	24.3
high	male	lamb	2005	31	twin	199	15.9	43.5

high	male	lamb	2005	31	twin	199	13.3	42.2
high	female	lamb	2005	32	twin	231	26.5	27.9
high	female	lamb	2005	32	twin	231	27.6	26.3
high	male	lamb	2005	32	twin	231	13	63.2
high	male	lamb	2005	32	twin	231	13.1	65.5
low	male	lamb	2005	32	twin	198	23.7	32.1
low	male	lamb	2005	32	twin	198	22.3	33.5
high	female	lamb	2005	32	asingleton	215	27.5	15.1
high	female	lamb	2005	32	asingleton	215	27.6	20.2
low	female	lamb	2005	32	triplet	231	34.6	40.6
low	female	lamb	2005	32	triplet	231	37.3	38.6
low	male	lamb	2005	33	asingleton	200	27.4	38.2
low	male	lamb	2005	33	asingleton	200	27.3	40.3
high	female	lamb	2005	33	asingleton	201	37.3	3.6
high	female	lamb	2005	33	asingleton	201	40.6	4.7
low	male	lamb	2005	33	twin	226	47.6	22.4
low	male	lamb	2005	33	twin	226	46.4	24.5
high	female	lamb	2005	34	twin	216	32.4	17.8
high	female	lamb	2005	34	twin	216	34.3	19.5
high	male	lamb	2005	34	twin	226	37.2	22.9
high	male	lamb	2005	34	twin	226	36.4	24.1
high	female	lamb	2005	35	triplet	231	27.6	25.7
high	female	lamb	2005	35	triplet	231	28.2	26
high	male	lamb	2005	37	twin	215	16.7	35.1
high	male	lamb	2005	37	twin	215	13.8	36.7
low	female	lamb	2005	37	triplet	227	43.9	35
low	female	lamb	2005	37	triplet	227	45.5	36.5
low	male	lamb	2005	39	asingleton	215	35.6	16.8
low	male	lamb	2005	39	asingleton	215	35.3	16.9
low	female	lamb	2005	40	asingleton	227	35.4	34.2
low	female	lamb	2005	40	asingleton	227	37.4	35.9
high	male	lamb	2005	41	twin	226	39.7	8.8
high	male	lamb	2005	41	twin	226	40.6	9.2
low	female	lamb	2005	41	triplet	215	24.3	36.7
low	female	lamb	2005	41	triplet	215	23.5	34.8
high	male	lamb	2005	43	twin	226	35.9	11
high	male	lamb	2005	43	twin	226	35	11.1
low	NA	lamb	2005	45	asingleton	215	28.3	41.7
low	NA	lamb	2005	45	asingleton	215	29.3	41.9
low	female	lamb	2005	46	triplet	227	37.1	31.9
low	female	lamb	2005	46	triplet	227	34.7	34.8
low	male	lamb	2005	46	asingleton	199	18.6	38.8
low	male	lamb	2005	46	asingleton	199	14.4	39.1
low	NA	lamb	2005	48	asingleton	227	43.5	39.1
low	NA	lamb	2005	48	asingleton	227	42.7	38.6
low	male	lamb	2005	59	asingleton	228	32.9	41.9
low	male	lamb	2005	59	asingleton	228	30.1	39.3
low	NA	ewe	2005	63	asingleton	186	29.7	36
low	NA	ewe	2005	63	asingleton	186	28.3	36.9
low	NA	ewe	2005	66	asingleton	215	25.9	24.4
low	NA	ewe	2005	66	asingleton	215	26.7	28.5
low	NA	ewe	2005	67	asingleton	226	22.3	38.1
low	NA	ewe	2005	67	asingleton	226	24.9	36.7
low	NA	ewe	2005	71	asingleton	200	25.4	37.5
low	NA	ewe	2005	71	asingleton	200	23.3	36
low	NA	ewe	2005	73	asingleton	216	21.4	20.2

low	NA	ewe	2005	73	asingleton	216	18.5	19.9
high	NA	ewe	2005	73	triplet	198	26.8	35.1
high	NA	ewe	2005	73	triplet	198	25	34.5
high	NA	ewe	2005	74	triplet	201	24.5	31.6
high	NA	ewe	2005	74	triplet	201	25.9	28.2
low	NA	ewe	2005	75	asingleton	226	26.5	15.7
low	NA	ewe	2005	75	asingleton	226	25.4	14.4
high	NA	ewe	2005	75	twin	189	21.7	37.4
high	NA	ewe	2005	75	twin	189	21.2	38.3
high	NA	ewe	2005	75	twin	189	45.9	13.1
high	NA	ewe	2005	75	twin	189	44.3	12.3
high	NA	ewe	2005	79	triplet	215	26.5	15.2
high	NA	ewe	2005	79	triplet	215	25.8	14.2
low	NA	ewe	2005	80	twin	200	27	45.6
low	NA	ewe	2005	80	twin	200	24.2	41.9
high	NA	ewe	2005	81	twin	228	73.2	5.6
high	NA	ewe	2005	81	twin	228	74.8	7
high	NA	ewe	2005	83	asingleton	228	61.2	4.7
high	NA	ewe	2005	83	asingleton	228	57.8	8.1
high	NA	ewe	2005	84	triplet	195	36.5	12.3
high	NA	ewe	2005	84	triplet	195	33.5	13.6
high	NA	ewe	2005	84	asingleton	201	46.2	5.7
high	NA	ewe	2005	84	asingleton	201	40.2	6.8
high	NA	ewe	2005	85	asingleton	187	26.2	20.2
high	NA	ewe	2005	85	asingleton	187	27.7	20.4
low	NA	ewe	2005	86	asingleton	188	22	52.8
low	NA	ewe	2005	86	asingleton	188	19.7	51.5
low	NA	ewe	2005	86	twin	231	46.5	12.3
low	NA	ewe	2005	86	twin	231	45.9	14.9
high	NA	ewe	2005	86	asingleton	197	19.9	19
high	NA	ewe	2005	86	asingleton	197	20.4	19.9
low	NA	ewe	2005	87	triplet	191	30.1	36.6
low	NA	ewe	2005	87	triplet	191	30.9	35
low	NA	ewe	2005	87	asingleton	187	40.8	28.1
low	NA	ewe	2005	87	asingleton	187	38.7	25.3
high	NA	ewe	2005	89	twin	186	29.2	33.2
high	NA	ewe	2005	89	twin	186	28.5	33.9
high	NA	ewe	2005	91	twin	227	17.7	34
high	NA	ewe	2005	91	twin	227	16.5	36
high	NA	ewe	2005	91	twin	215	17	41
high	NA	ewe	2005	91	twin	215	16.7	42.6
high	NA	ewe	2005	92	twin	200	21	23.8
high	NA	ewe	2005	92	twin	200	19.2	23.6
low	NA	ewe	2005	92	triplet	201	47	10.3
low	NA	ewe	2005	92	triplet	201	45.7	10
low	NA	ewe	2005	92	asingleton	227	48.1	28
low	NA	ewe	2005	92	asingleton	227	48.3	25.8
high	NA	ewe	2005	93	twin	228	22.1	18.3
high	NA	ewe	2005	93	twin	228	20.7	18.8
low	NA	ewe	2005	93	triplet	228	51.4	7.9
low	NA	ewe	2005	93	triplet	228	53	10.7
low	NA	ewe	2005	94	triplet	216	31.2	34.4
low	NA	ewe	2005	94	triplet	216	34	33.5
low	NA	ewe	2005	95	asingleton	228	44.8	26.2
low	NA	ewe	2005	95	asingleton	228	47.5	27.1
high	NA	ewe	2005	96	asingleton	201	16	29.6

high	NA	ewe	2005	96	asingleton	201	16.2	31.5
high	male	lamb	2006	15	triplet	185	8.5	57.7
high	male	lamb	2006	15	triplet	185	9.2	59.5
low	female	lamb	2006	16	triplet	188	23.8	45.6
low	female	lamb	2006	16	triplet	188	23.0	46.3
low	male	lamb	2006	16	triplet	185	24.6	32.4
low	male	lamb	2006	16	triplet	185	25.3	32.8
low	female	lamb	2006	17	triplet	202	5.5	63.3
low	female	lamb	2006	17	triplet	202	8.1	64.7
low	male	lamb	2006	20	triplet	200	15.0	51.5
low	male	lamb	2006	20	triplet	200	13.3	51.3
low	female	lamb	2006	21	triplet	207	20.1	33.2
low	female	lamb	2006	21	triplet	207	19.6	34.7
low	male	lamb	2006	21	triplet	201	10.6	54.0
low	male	lamb	2006	21	triplet	201	11.9	56.7
low	male	lamb	2006	22	triplet	205	14.8	59.2
low	male	lamb	2006	22	triplet	205	14.0	61.6
high	female	lamb	2006	22	asingleton	199	21.7	13.8
high	female	lamb	2006	22	asingleton	199	22.9	12.4
low	male	lamb	2006	22	asingleton	180	12.7	31.7
low	male	lamb	2006	22	asingleton	180	13.5	31.1
high	female	lamb	2006	23	twin	236	69.4	12.0
high	female	lamb	2006	23	twin	236	67.8	13.2
low	male	lamb	2006	24	twin	179	14.0	28.8
low	male	lamb	2006	24	twin	179	14.2	28.3
high	female	lamb	2006	24	twin	193	5.2	14.0
high	female	lamb	2006	24	twin	193	6.1	15.2
high	female	lamb	2006	28	twin	205	20.4	19.6
high	female	lamb	2006	28	twin	205	18.8	21.5
low	male	lamb	2006	28	twin	208	8.7	70.2
low	male	lamb	2006	28	twin	208	7.5	69.6
high	NA	lamb	2006	28	triplet	208	17.7	39.8
high	NA	lamb	2006	28	triplet	208	18.1	41.0
low	female	lamb	2006	29	asingleton	193	16.8	39.6
low	female	lamb	2006	29	asingleton	193	16.1	37.9
low	male	lamb	2006	29	twin	215	32.1	50.5
low	male	lamb	2006	29	twin	215	31.7	52.7
low	male	lamb	2006	29	asingleton	199	30.0	18.4
low	male	lamb	2006	29	asingleton	199	28.9	19.9
high	female	lamb	2006	30	twin	214	20.5	13.0
high	female	lamb	2006	30	twin	214	20.2	13.4
high	female	lamb	2006	31	twin	214	5.9	7.6
high	female	lamb	2006	31	twin	214	5.8	9.3
low	male	lamb	2006	31	asingleton	204	20.7	26.3
low	male	lamb	2006	31	asingleton	204	22.1	27.5
high	female	lamb	2006	31	twin	214	45.0	7.9
high	female	lamb	2006	31	twin	214	47.0	10.5
low	male	lamb	2006	32	asingleton	192	28.4	35.6
low	male	lamb	2006	32	asingleton	192	26.6	37.1
high	male	lamb	2006	32	triplet	235	40.1	18.1
high	male	lamb	2006	32	triplet	235	39.1	18.4
high	female	lamb	2006	32	asingleton	235	36.3	14.3
high	female	lamb	2006	32	asingleton	235	37.3	11.8
high	female	lamb	2006	33	asingleton	236	49.1	14.4
high	female	lamb	2006	33	asingleton	236	51.0	14.0
high	male	lamb	2006	33	twin	209	49.0	17.8

high	male	lamb	2006	33	twin	209	50.2	17.1
low	male	lamb	2006	35	asingleton	185	18.6	23.3
low	male	lamb	2006	35	asingleton	185	20.1	21.5
high	female	lamb	2006	35	asingleton	188	29.7	27.1
high	female	lamb	2006	35	asingleton	188	27.8	29.5
high	male	lamb	2006	36	twin	234	24.2	22.0
high	male	lamb	2006	36	twin	234	26.4	21.7
high	female	lamb	2006	37	twin	235	24.2	22.0
high	female	lamb	2006	37	twin	235	26.4	21.7
high	female	lamb	2006	38	twin	233	56.6	10.7
high	female	lamb	2006	38	twin	233	57.6	10.0
high	female	lamb	2006	39	asingleton	200	41.6	22.8
high	female	lamb	2006	39	asingleton	200	39.7	23.0
low	male	lamb	2006	39	twin	224	28.8	28.8
low	male	lamb	2006	39	twin	224	28.6	31.3
low	male	lamb	2006	39	asingleton	214	22.6	33.5
low	male	lamb	2006	39	asingleton	214	25.1	31.6
low	male	lamb	2006	43	twin	235	51.8	19.2
low	male	lamb	2006	43	twin	235	52.3	17.9
low	male	lamb	2006	46	asingleton	235	52.0	11.3
low	male	lamb	2006	46	asingleton	235	50.4	12.0
low	NA	lamb	2007	16	twin	180	0.0	42.6
high	male	lamb	2007	17	twin	180	32.8	29.8
low	male	lamb	2007	19	triplet	186	22.2	35.8
high	female	lamb	2007	19	triplet	192	27.5	29.3
low	NA	lamb	2007	19	twin	187	9.9	56.3
low	female	lamb	2007	20	asingleton	192	18.8	33.9
low	male	lamb	2007	21	triplet	187	11.6	30.4
low	male	lamb	2007	21	twin	192	15.9	52.2
high	male	lamb	2007	21	twin	197	13.9	40.7
high	male	lamb	2007	22	twin	187	29.7	24.1
high	male	lamb	2007	22	twin	199	16.5	17.9
low	female	lamb	2007	23	triplet	201	14.1	37.5
low	NA	lamb	2007	24	twin	201	14.1	43.3
high	NA	lamb	2007	25	triplet	219	15.7	42.5
low	male	lamb	2007	26	triplet	193	42.3	19.4
low	NA	lamb	2007	26	twin	213	18.9	27.6
high	female	lamb	2007	27	triplet	228	47.8	14.7
high	NA	lamb	2007	29	twin	212	34.7	15.4
low	male	lamb	2007	29	triplet	201	19	35
high	male	lamb	2007	29	twin	200	28.9	31.3
low	female	lamb	2007	31	triplet	226	15.4	35.6
high	male	lamb	2007	31	twin	197	41.8	7.8
low	male	lamb	2007	31	twin	222	23.4	29.4
high	female	lamb	2007	31	twin	240	49.6	12.4
high	NA	lamb	2007	31	twin	222	43	11.3
low	male	lamb	2007	32	twin	214	40	22.8
high	male	lamb	2007	32	asingleton	199	11.6	19.2
low	male	lamb	2007	32	twin	226	36	18.1
high	male	lamb	2007	32	twin	228	22.8	13.8
low	male	lamb	2007	33	triplet	219	18.8	35.6
low	male	lamb	2007	33	twin	219	27.4	30.9
high	NA	lamb	2007	34	asingleton	222	55	11.8
high	male	lamb	2007	37	twin	240	56.1	14
high	female	lamb	2007	37	triplet	234	50	14.8
high	male	lamb	2007	37	twin	219	40.4	1.8

high	male	lamb	2007	37	twin	235	10.5	6
high	male	lamb	2007	38	triplet	228	28.1	26.7
high	male	lamb	2007	38	twin	234	68.5	3.3
low	male	lamb	2007	42	triplet	235	12.3	47.3
low	male	lamb	2007	49	asingleton	234	19.5	43.9
low	male	lamb	2007	52	asingleton	240	34.6	29.4
high	male	lamb	2008	17	triplet	202	29.3	37.8
low	male	lamb	2008	17	triplet	181	17	49.2
high	female	lamb	2008	18	triplet	184	13.3	19.8
low	female	lamb	2008	19	twin	191	21.5	11
low	male	lamb	2008	19	triplet	196	12.8	46.9
low	female	lamb	2008	20	asingleton	196	10.1	46.4
low	male	lamb	2008	20	twin	189	11.1	27.8
low	female	lamb	2008	20	asingleton	198	5.5	42
high	female	lamb	2008	20	triplet	191	18.4	12.9
high	female	lamb	2008	22	triplet	190	20.7	19.2
high	male	lamb	2008	22	twin	203	19.1	14.6
high	female	lamb	2008	22	twin	191	11.7	23.7
low	female	lamb	2008	22	twin	185	12.4	30.8
high	female	lamb	2008	22	asingleton	183	18.4	18.8
low	female	lamb	2008	23	twin	204	9.4	44.7
low	male	lamb	2008	24	triplet	211	12.2	33.8
high	male	lamb	2008	24	asingleton	195	32.6	15.3
low	male	lamb	2008	25	twin	204	4.2	30.1
low	male	lamb	2008	25	twin	184	14	33.7
high	male	lamb	2008	26	triplet	191	18.7	6.3
high	male	lamb	2008	27	asingleton	195	10.1	11
low	female	lamb	2008	27	twin	198	3.6	45.9
high	male	lamb	2008	28	triplet	198	14.6	15.2
high	female	lamb	2008	29	asingleton	198	25	29.2
high	female	lamb	2008	29	twin	233	40.6	8.2
high	female	lamb	2008	29	twin	203	13.2	12.9
high	male	lamb	2008	29	asingleton	203	11.9	18.7
low	NA	lamb	2008	31	asingleton	191	15.9	39
low	male	lamb	2008	31	twin	195	10.1	33.5
high	NA	lamb	2008	31	asingleton	196	10.7	17.1
low	male	lamb	2008	31	triplet	231	34.4	14.3
low	female	lamb	2008	32	twin	232	52.2	19.4
high	female	lamb	2008	32	twin	226	24.2	15.3
high	NA	lamb	2008	32	asingleton	198	4.3	25.7
low	male	lamb	2008	33	twin	203	9	47.6
low	female	lamb	2008	33	asingleton	202	13.5	35
low	female	lamb	2008	33	asingleton	199	12.4	33.6
high	male	lamb	2008	34	triplet	241	44.1	17.1
low	male	lamb	2008	34	twin	231	13.5	21.2
high	male	lamb	2008	35	asingleton	202	22.8	16.1
high	female	lamb	2008	36	twin	232	44.6	12.5
high	male	lamb	2008	36	twin	245	44.6	11.4
high	male	lamb	2008	37	asingleton	241	19.2	15.4
low	female	lamb	2008	37	twin	226	12.1	39.2
high	female	lamb	2008	41	twin	241	42.3	7.8
high	female	lamb	2008	41	asingleton	245	43.6	10.3
low	male	lamb	2008	44	twin	245	15.9	26.6
low	female	lamb	2008	44	twin	245	29	40.8
low	NA	lamb	2008	46	asingleton	232	40.7	16.7
low	male	lamb	2008	47	twin	230	31	18.9

low	female	lamb	2008	47	asingleton	237	46.3	29.4
high	NA	lamb	2008	48	asingleton	245	36.3	12.4
high	NA	ewe	2008	51	asingleton	183	37.4	10.9
high	NA	ewe	2008	52	asingleton	210	18.1	12.4
low	NA	ewe	2008	54	asingleton	184	42.8	19.1
high	NA	ewe	2008	55	twin	184	33.1	13.9
high	NA	ewe	2008	55	asingleton	196	30	16.9
high	NA	ewe	2008	56	twin	198	17.7	15.1
low	NA	ewe	2008	56	asingleton	198	12.8	57.5
high	NA	ewe	2008	57	asingleton	232	53.6	9.5
high	NA	ewe	2008	57	twin	198	17.5	18.6
high	NA	ewe	2008	59	asingleton	195	22.4	12.3
high	NA	ewe	2008	60	asingleton	202	28	11.2
high	NA	ewe	2008	60	asingleton	241	84.2	3.8
low	NA	ewe	2008	62	asingleton	233	37.9	9.8
high	NA	ewe	2008	62	twin	203	16.2	15.2
high	NA	ewe	2008	64	asingleton	191	23.7	13.3
high	NA	ewe	2008	64	asingleton	238	30.3	8.2
low	NA	ewe	2008	66	asingleton	195	25.1	24.6
low	NA	ewe	2008	66	asingleton	202	7.9	40
high	NA	ewe	2008	67	asingleton	212	22.6	15
high	NA	ewe	2008	68	twin	241	49.3	7.1
low	NA	ewe	2008	68	asingleton	241	44.7	17.9
high	NA	ewe	2008	69	twin	202	20.7	9.6
high	NA	ewe	2008	69	asingleton	246	49	5.8
high	NA	ewe	2008	76	triplet	190	30.9	14.3
high	NA	ewe	2008	76	triplet	233	61.1	7.2
high	NA	ewe	2008	77	twin	210	14.1	12.6
high	NA	ewe	2008	79	twin	210	46.4	8.8
high	NA	ewe	2008	79	asingleton	246	58.8	7.1
high	NA	ewe	2008	80	asingleton	185	32.9	27.1
low	NA	ewe	2008	80	twin	247	48.4	18
low	NA	ewe	2008	81	twin	231	46.2	23.2
low	NA	ewe	2008	81	twin	210	22	24.4
low	NA	ewe	2008	81	twin	196	16.8	24.2
low	NA	ewe	2008	81	twin	198	15	42.7
low	NA	ewe	2008	81	twin	198	22.2	34.8
low	NA	ewe	2008	82	twin	183	25.6	38.4
low	NA	ewe	2008	82	twin	181	25.2	21.6
low	NA	ewe	2008	83	twin	231	28.4	24.4
low	NA	ewe	2008	84	twin	183	29	21.2
low	NA	ewe	2008	84	twin	195	26.1	17.9
low	NA	ewe	2008	84	twin	211	18.6	15.9
low	NA	ewe	2008	84	twin	225	27.9	14.9
low	NA	ewe	2008	84	twin	240	50.5	19.6
high	NA	ewe	2008	87	twin	185	40.2	13.5
high	NA	ewe	2008	87	twin	203	27.6	18.8
high	NA	ewe	2008	87	twin	233	48.7	4.9
low	NA	ewe	2008	91	asingleton	191	30	20.6
low	NA	ewe	2008	91	asingleton	198	14.8	24.3
low	NA	ewe	2008	91	asingleton	237	42.4	18.2
high	NA	ewe	2008	91	triplet	231	27.9	14.3
high	NA	ewe	2008	92	asingleton	189	31.5	18.9
high	NA	ewe	2008	93	triplet	198	39.5	19.7
low	NA	ewe	2008	95	triplet	181	28.3	26.2
low	NA	ewe	2008	95	triplet	202	9.7	30.2

low	NA	ewe	2008	95	triplet	231	44.3	26.5
high	NA	ewe	2008	97	triplet	191	33.5	6.9
high	NA	ewe	2008	97	triplet	228	37.9	10
high	NA	ewe	2008	110	triplet	183	32.2	18.4
high	NA	lamb	2009	17	triplet	191	4.1	34.7
high	male	lamb	2009	20	triplet	193	17.7	17.3
low	male	lamb	2009	21	triplet	180	6.9	40.5
low	female	lamb	2009	21	triplet	194	17.2	26.6
high	male	lamb	2009	21	twin	191	4.9	42.8
high	female	lamb	2009	21	triplet	205	35.8	6.2
high	female	lamb	2009	21	triplet	205	34.7	11
high	female	lamb	2009	22	twin	195	23.3	26.3
high	male	lamb	2009	23	twin	195	34.7	30.7
high	male	lamb	2009	23	triplet	194	13.3	26.1
high	female	lamb	2009	23	twin	194	1.9	15.5
high	male	lamb	2009	23	triplet	214	29	18.8
high	male	lamb	2009	24	twin	193	14.6	21.5
high	female	lamb	2009	24	twin	194	11.3	24.6
high	female	lamb	2009	24	twin	193	5.7	6.4
low	NA	lamb	2009	25	triplet	194	10.2	51.5
high	male	lamb	2009	25	triplet	194	29.3	22.1
low	female	lamb	2009	25	asingleton	184	10.6	27.8
high	male	lamb	2009	25	triplet	233	39.2	15.9
low	male	lamb	2009	26	twin	191	9.3	39.9
low	female	lamb	2009	26	triplet	218	52	36.5
low	NA	lamb	2009	26	asingleton	188	8.2	38.4
low	male	lamb	2009	26	twin	190	7.4	32.1
high	female	lamb	2009	27	twin	233	31.6	16.6
high	male	lamb	2009	27	twin	193	10.3	8.3
high	male	lamb	2009	29	twin	217	29.9	16.8
high	female	lamb	2009	29	twin	224	34	28.3
low	male	lamb	2009	29	triplet	213	10.6	60.6
high	male	lamb	2009	29	triplet	226	40.3	13.8
low	male	lamb	2009	29	asingleton	198	13.7	29.1
low	female	lamb	2009	30	triplet	220	44.2	22.4
low	male	lamb	2009	30	twin	202	8.4	54.1
high	female	lamb	2009	30	twin	218	37.7	14.2
high	male	lamb	2009	30	twin	230	53.6	14.3
low	male	lamb	2009	30	twin	202	12.6	39.1
high	male	lamb	2009	31	twin	227	55.5	20.3
high	female	lamb	2009	31	asingleton	229	34.1	16.2
low	female	lamb	2009	32	twin	218	23.9	38.7
low	female	lamb	2009	33	asingleton	205	13.5	49.4
high	male	lamb	2009	33	triplet	225	39.9	25.5
high	male	lamb	2009	34	asingleton	218	24.9	13
high	male	lamb	2009	34	twin	233	65.5	13.2
high	female	lamb	2009	35	twin	223	39.5	15.7
high	female	lamb	2009	35	twin	235	38.5	14.7
low	male	lamb	2009	35	twin	220	38.8	32.1
low	NA	lamb	2009	35	triplet	224	45.2	26
high	male	lamb	2009	35	triplet	218	33	28.2
low	male	lamb	2009	36	triplet	218	36.8	30.1
high	male	lamb	2009	37	triplet	239	45.5	15.2
high	male	lamb	2009	37	twin	218	28.4	15.3
low	NA	lamb	2009	38	asingleton	218	22.6	41.4
low	female	lamb	2009	38	asingleton	220	37.5	30.4

low	female	lamb	2009	39	twin	236	40.6	26.4
low	male	lamb	2009	40	asingleton	223	23.2	43.3
low	male	lamb	2009	40	twin	223	50.5	17.1
high	female	lamb	2009	40	asingleton	233	33.1	28.3
low	NA	ewe	2009	61	triplet	180	14.4	58.7
high	NA	ewe	2009	64	twin	190	21.5	22.8
low	NA	ewe	2009	64	triplet	205	25	29.3
high	NA	ewe	2009	65	twin	228	33.6	19.4
high	NA	ewe	2009	65	twin	233	48.8	12.2
low	NA	ewe	2009	66	triplet	226	27.3	44.9
high	NA	ewe	2009	66	twin	233	55.7	10.4
high	NA	ewe	2009	67	twin	197	7.9	24.6
high	NA	ewe	2009	68	twin	182	19.4	9.8
high	NA	ewe	2009	68	twin	214	42.4	12.8
low	NA	ewe	2009	68	triplet	227	33.7	8.3
low	NA	ewe	2009	69	triplet	202	27.3	24.8
high	NA	ewe	2009	69	twin	226	46.9	12.4
low	NA	ewe	2009	69	triplet	191	16.1	14.7
high	NA	ewe	2009	70	twin	218	50.8	10.1
high	NA	ewe	2009	70	asingleton	229	62.2	9.8
high	NA	ewe	2009	71	triplet	214	37.1	23.9
low	NA	ewe	2009	72	asingleton	190	26.4	25.8
high	NA	ewe	2009	72	twin	188	24.3	13.4
low	NA	ewe	2009	72	asingleton	202	31.2	26
low	NA	ewe	2009	72	asingleton	223	57.4	12.9
high	NA	ewe	2009	73	triplet	220	56.3	9
high	NA	ewe	2009	74	triplet	193	16.6	16.2
high	NA	ewe	2009	74	twin	205	40.3	15.8
high	NA	ewe	2009	74	twin	193	29.3	13.8
high	NA	ewe	2009	75	triplet	205	34.8	10.1
high	NA	ewe	2009	75	twin	224	50.4	5.3
high	NA	ewe	2009	76	twin	183	28.8	19.4
high	NA	ewe	2009	76	twin	219	51.8	10.4
low	NA	ewe	2009	76	asingleton	230	33.5	17.6
low	NA	ewe	2009	76	twin	220	42	23.2
low	NA	ewe	2009	76	asingleton	220	42.6	17.9
high	NA	ewe	2009	76	triplet	227	47.2	17.1
high	NA	ewe	2009	76	triplet	225	43.3	29.1
high	NA	ewe	2009	76	twin	183	12.2	14.9
low	NA	ewe	2009	76	twin	199	36.1	20.9
low	NA	ewe	2009	76	twin	194	26.6	26.1
low	NA	ewe	2009	76	asingleton	177	36.8	27.2
high	NA	ewe	2009	77	triplet	198	15.2	30.2
high	NA	ewe	2009	77	triplet	191	29.3	25.5
high	NA	ewe	2009	77	triplet	191	7.9	36.3
high	NA	ewe	2009	77	asingleton	183	9.1	18.1
high	NA	ewe	2009	77	asingleton	197	7.5	23.8
high	NA	ewe	2009	77	twin	183	45.3	23.4
high	NA	ewe	2009	77	triplet	179	22.6	17.4
low	NA	ewe	2009	79	triplet	188	21.3	21.1
low	NA	ewe	2009	79	asingleton	180	16.3	38.9
high	NA	ewe	2009	80	twin	223	72.5	9.5
high	NA	ewe	2009	80	twin	229	29	23.4
low	NA	ewe	2009	80	triplet	199	26.5	20.3
low	NA	ewe	2009	84	asingleton	198	23.7	24.1
low	NA	ewe	2009	84	triplet	224	38.6	19.9

high	NA	ewe	2009	86	asingleton	232	50.9	17
low	NA	ewe	2009	91	asingleton	225	47.4	18.9
high	male	lamb	2010	16	twin	210	16.9	9.1
high	male	lamb	2010	18	triplet	181	31.1	33.3
high	male	lamb	2010	19	twin	182	16.3	41
high	male	lamb	2010	19	triplet	216	30.4	46.5
high	male	lamb	2010	20	triplet	232	59.1	11
high	male	lamb	2010	20	triplet	193	17.5	41.2
high	male	lamb	2010	21	triplet	216	49.3	9.8
high	male	lamb	2010	21	twin	216	26.3	7.3
low	male	lamb	2010	21	triplet	188	16.6	34.2
high	male	lamb	2010	22	twin	191	13	25.5
low	male	lamb	2010	22	triplet	193	22.7	35.9
low	male	lamb	2010	22	triplet	189	33.5	30.5
low	male	lamb	2010	22	triplet	193	43.1	21.1
high	male	lamb	2010	22	twin	194	7.1	6.9
high	male	lamb	2010	22	twin	188	14.7	42.8
high	male	lamb	2010	23	triplet	230	59.5	15.5
high	male	lamb	2010	24	twin	195	12.7	33.3
high	male	lamb	2010	25	twin	191	11.9	34
high	male	lamb	2010	25	twin	230	58.4	6.7
high	male	lamb	2010	25	twin	204	17.6	7.2
low	male	lamb	2010	26	triplet	205	15.1	45.6
low	male	lamb	2010	27	triplet	214	27.1	38.9
high	male	lamb	2010	28	twin	210	23.1	38.8
high	male	lamb	2010	28	triplet	214	13.7	42.6
low	female	lamb	2010	28	twin	189	21.9	32.4
low	female	lamb	2010	28	triplet	207	28.8	32.2
low	male	lamb	2010	29	triplet	207	25.3	46.6
low	male	lamb	2010	29	twin	204	27.2	32
high	male	lamb	2010	31	triplet	234	49	22.5
low	male	lamb	2010	31	asingleton	183	23.1	38.4
high	male	lamb	2010	31	twin	236	59	13.3
low	male	lamb	2010	32	triplet	215	33.7	28.4
low	male	lamb	2010	33	triplet	235	35.9	16.5
high	male	lamb	2010	34	twin	228	40	18.8
high	male	lamb	2010	35	twin	237	41.7	12.9
low	male	lamb	2010	35	triplet	228	36.3	34.2
low	female	lamb	2010	37	asingleton	191	12.9	28.6
low	male	lamb	2010	37	triplet	235	45.2	34.9
low	male	lamb	2010	37	triplet	207	30.1	26.2
low	male	lamb	2010	37	asingleton	195	10.1	45.5
low	female	lamb	2010	37	triplet	237	38.9	26.7
low	female	lamb	2010	38	triplet	234	53.7	17.6
high	male	lamb	2010	39	twin	236	54.5	20.9
low	male	lamb	2010	39	twin	237	38.4	23.3
high	male	lamb	2010	40	twin	237	60.8	7.1
low	male	lamb	2010	41	triplet	237	44.5	25.2
high	male	lamb	2010	42	asingleton	207	24.1	21
high	male	lamb	2010	46	twin	237	47.9	7.9
low	male	lamb	2010	57	asingleton	237	23.1	47.8
high	NA	ewe	2010	68	triplet	230	32.7	21.2
high	NA	ewe	2010	71	twin	209	16.1	50.8
high	NA	ewe	2010	73	twin	191	41.2	5.6
high	NA	ewe	2010	73	twin	230	63.5	12
high	NA	ewe	2010	74	twin	204	14.1	10

high	NA	ewe	2010	74	twin	236	53.8	15.7
high	NA	ewe	2010	76	twin	188	26.5	24.2
high	NA	ewe	2010	79	twin	183	23.5	10.4
high	NA	ewe	2010	79	twin	210	36.6	20
high	NA	ewe	2010	80	twin	237	82.2	4.6
high	NA	ewe	2010	80	twin	227	67.3	13.7
high	NA	ewe	2010	80	twin	193	18.1	21.8
low	NA	ewe	2010	80	triplet	233	49.1	21.9
low	NA	ewe	2010	81	triplet	189	31.9	29.6
low	NA	ewe	2010	81	triplet	235	16.6	33.3
low	NA	ewe	2010	82	triplet	189	33.8	27.9
low	NA	ewe	2010	82	triplet	180	39.1	24.4
low	NA	ewe	2010	82	asingleton	183	37.8	35.8
low	NA	ewe	2010	82	twin	182	26.8	31.5
low	NA	ewe	2010	82	triplet	228	50.5	15.8
low	NA	ewe	2010	82	twin	189	0	0
low	NA	ewe	2010	83	triplet	237	52.8	12.4
low	NA	ewe	2010	83	triplet	214	44.8	27.8
low	NA	ewe	2010	83	asingleton	195	23.8	36.7
low	NA	ewe	2010	84	triplet	203	24.2	30.8
high	NA	ewe	2010	84	twin	232	62.5	5.1
low	NA	ewe	2010	85	asingleton	215	47.6	19.1
high	NA	ewe	2010	85	triplet	236	52.9	15.6
low	NA	ewe	2010	85	triplet	191	26.1	32.1
high	NA	ewe	2010	86	triplet	216	49.2	23.1
low	NA	ewe	2010	87	asingleton	230	46.5	29.9
high	NA	ewe	2010	87	twin	194	22.2	5.4
high	NA	ewe	2010	89	twin	195	23.1	17
low	NA	ewe	2010	90	asingleton	207	39	14.3
low	NA	ewe	2010	90	asingleton	216	46.1	17.4
high	NA	ewe	2010	90	twin	181	32.2	20.3
high	NA	ewe	2010	92	twin	216	26.4	3.3
high	NA	ewe	2010	94	triplet	237	71.6	6.9
high	NA	ewe	2010	95	triplet	193	27.8	18.2
high	NA	ewe	2010	96	twin	189	31.1	7.2
high	NA	ewe	2010	101	twin	207	47.2	13.2
high	NA	ewe	2010	113	asingleton	236	52.2	10
low	female	lamb	2011	14	triplet	185	25.6	32.3
high	male	lamb	2011	16	twin	192	5.8	34.3
low	male	lamb	2011	17	triplet	211	31.4	31.2
high	male	lamb	2011	17	triplet	188	19.3	35.4
low	male	lamb	2011	18	triplet	190	21.8	30.6
high	male	lamb	2011	19	twin	200	17.3	6.9
high	male	lamb	2011	19	twin	185	24.6	10.4
low	male	lamb	2011	19	triplet	186	14.9	32.6
high	male	lamb	2011	20	triplet	181	16.3	34.9
low	male	lamb	2011	20	triplet	199	23.3	19.7
high	male	lamb	2011	21	triplet	188	22.1	13.1
low	male	lamb	2011	21	triplet	211	35.4	33.3
low	male	lamb	2011	21	triplet	235	57.4	22.7
low	female	lamb	2011	22	triplet	181	32.9	23.8
high	male	lamb	2011	22	twin	192	25.4	16.4
high	male	lamb	2011	22	triplet	215	35.4	17
high	male	lamb	2011	22	triplet	213	49.4	26
low	male	lamb	2011	23	triplet	209	29	33.1
high	male	lamb	2011	23	twin	185	27.2	13.8

low	male	lamb	2011	23	twin	185	14.6	48.6
high	female	lamb	2011	23	twin	181	41.5	7.1
low	male	lamb	2011	24	twin	190	10.2	46.2
low	female	lamb	2011	25	triplet	211	23.7	21.6
low	male	lamb	2011	25	triplet	229	48.9	25.3
high	male	lamb	2011	25	twin	213	43	18.2
low	male	lamb	2011	26	twin	205	35.8	20.8
high	male	lamb	2011	26	twin	193	28.3	11
low	male	lamb	2011	26	triplet	213	47.8	13.3
high	male	lamb	2011	27	triplet	192	34.9	16.7
low	male	lamb	2011	27	triplet	214	46.6	15
high	male	lamb	2011	28	twin	213	47.4	16.7
high	male	lamb	2011	28	triplet	229	37.8	11.1
high	male	lamb	2011	28	triplet	211	35.9	15.9
high	male	lamb	2011	29	twin	204	25.5	35
low	male	lamb	2011	29	triplet	238	44.8	14.6
high	male	lamb	2011	29	triplet	218	36.6	7.2
low	male	lamb	2011	29	triplet	235	46.1	32.4
high	male	lamb	2011	30	twin	218	62.3	7.5
low	male	lamb	2011	31	twin	221	45.8	31.8
high	male	lamb	2011	31	twin	214	26.2	7.8
high	female	lamb	2011	31	twin	210	22.6	13.1
low	male	lamb	2011	31	twin	204	25.4	27.9
low	male	lamb	2011	31	twin	211	43.3	22.6
low	female	lamb	2011	32	triplet	211	30	21.1
high	male	lamb	2011	33	triplet	210	10.5	38
low	female	lamb	2011	33	triplet	217	30.8	24.4
low	male	lamb	2011	34	twin	219	34.8	37
low	male	lamb	2011	34	asingleton	217	36.7	26.2
high	male	lamb	2011	34	triplet	233	25.7	6.4
high	male	lamb	2011	35	triplet	244	35.8	7.4
high	male	lamb	2011	35	twin	211	40.3	6.4
high	male	lamb	2011	35	twin	235	51.2	19.4
high	male	lamb	2011	35	twin	226	65.7	11
high	male	lamb	2011	37	twin	240	66.5	6.9
high	male	lamb	2011	37	twin	229	67.6	8.5
low	male	lamb	2011	38	twin	230	29.3	44.4
high	male	lamb	2011	39	twin	238	41.9	11.4
high	male	lamb	2011	40	twin	233	44.8	14.6
low	female	lamb	2011	40	triplet	240	50.4	13.4
low	female	lamb	2011	41	triplet	242	40.7	12.6
high	male	lamb	2011	41	twin	235	45.8	11.8
high	NA	ewe	2011	65	twin	184	23.1	16.2
high	NA	ewe	2011	66	twin	244	50	7
high	NA	ewe	2011	72	twin	218	79.1	4.8
high	NA	ewe	2011	72	twin	239	61.8	10.4
high	NA	ewe	2011	72	twin	239	74.7	5.7
high	NA	ewe	2011	72	twin	210	48	11.1
high	NA	ewe	2011	74	twin	218	69.3	8.6
low	NA	ewe	2011	74	triplet	185	36	26.6
high	NA	ewe	2011	74	twin	214	45.2	10.7
low	NA	ewe	2011	75	triplet	205	35.5	10.4
high	NA	ewe	2011	75	twin	218	67.5	5.9
high	NA	ewe	2011	75	twin	200	48.3	4.8
high	NA	ewe	2011	75	twin	209	50.9	11.8
low	NA	ewe	2011	75	triplet	223	56	10.9

high	NA	ewe	2011	76	twin	210	40.7	9.9
high	NA	ewe	2011	76	twin	190	32.6	14.8
high	NA	ewe	2011	76	triplet	221	35	9.5
high	NA	ewe	2011	77	twin	185	32	8.2
high	NA	ewe	2011	78	triplet	233	46.6	4.4
high	NA	ewe	2011	79	twin	209	35.5	14.8
high	NA	ewe	2011	79	twin	188	24.1	28.5
high	NA	ewe	2011	79	twin	185	38	6
high	NA	ewe	2011	80	twin	179	32.3	23.3
high	NA	ewe	2011	80	twin	192	26.6	22.3
high	NA	ewe	2011	80	twin	200	40.6	7.6
high	NA	ewe	2011	81	triplet	179	40.5	10.7
high	NA	ewe	2011	82	triplet	181	20.7	7.6
low	NA	ewe	2011	82	triplet	240	54.3	9.3
high	NA	ewe	2011	83	twin	210	27.2	11.1
high	NA	ewe	2011	84	twin	180	41.3	2.8
high	NA	ewe	2011	84	twin	179	46.1	4.7
high	NA	ewe	2011	84	triplet	222	59.4	6.7
low	NA	ewe	2011	85	triplet	209	45.8	18.4
low	NA	ewe	2011	88	triplet	184	34.3	18.6
low	NA	ewe	2011	91	twin	220	38.5	26.8
low	NA	ewe	2011	91	twin	202	23.2	38.6
high	NA	ewe	2011	91	triplet	205	53.7	3.8
low	NA	ewe	2011	91	twin	179	39.3	20.9
low	NA	ewe	2011	92	twin	209	46.9	15.4
low	NA	ewe	2011	92	triplet	204	35.2	22.6
high	NA	ewe	2011	92	triplet	190	27.7	5.1
low	NA	ewe	2011	92	triplet	179	39.5	21.3
high	NA	ewe	2011	93	triplet	180	36.8	6.5
low	NA	ewe	2011	93	asingleton	209	40.6	9.9
low	NA	ewe	2011	93	twin	184	35.3	26.9
high	NA	ewe	2011	99	asingleton	223	32.9	14.6
high	NA	ewe	2011	100	asingleton	213	38	10.4
